# Painful Knee is not Uncommon after total Knee Arthroplasty and can be Treated by Arthroscopic Debridement

**DOI:** 10.2174/1874325001711011147

**Published:** 2017-10-31

**Authors:** Hitoshi Sekiya

**Affiliations:** Shin-Kaminokawa Hospital - Orthopaedic Surgery, 2360 Kaminokawa Kaminokawa-machi Kawachi-gun, Tochigi Kaminokawa 329-0611, Japan

**Keywords:** Knee, Arthroplasty, Pain, Arthroscopy, Infrapatellar fat pad, Impingement, Patellar clunk syndrome, Patellar synovial hyperplasia

## Abstract

**Background:**

After total knee arthroplasty (TKA), most patients have an improvement; however, a few continue to have residual pain. We reported a case series of painful knee after TKA with unreported reason.

**Material and Methods:**

Forty-six arthroscopic surgeries were performed for painful knee after TKA. Of these, 16 were excluded due to infection, patellar clunk syndrome, patellofemoral synovial hyperplasia, aseptic loosening, or short follow up less than 6 months. Remaining 30 cases had marked tenderness at the medial and/or lateral tibiofemoral joint space, and they had pain during walking with pain or without pain at rest. The mean period from initial TKA to arthroscopy was 29 months, and the mean follow-up after arthroscopy was 36 months. All arthroscopic debridement was performed through 3 portals. Scar tissue impingements graded moderate or severe were found only in 30% of the cases in both the medial and lateral tibiofemoral joint spaces. The infrapatellar fat pad was covered with whitish scar tissue in all cases, and the tissue was connected with the scar tissue at the medial or lateral tibiofemoral joint spaces. All scar tissue was removed with a motorized shaver or punches.

**Results:**

At the final follow-up, 63% were pain free, 3% had marked improvement, 20% had half improvement, 3% had slight improvement, and 11% had no change. We hypothesized that the lesser mobility of the scar tissue due to the continuity of the tissue between the infrapatellar fat pad and the tibiofemoral joint space could cause easy impingement at the tibiofemoral joint, even with the small volume of scar tissue.

**Conclusion:**

If infection and aseptic loosening could be ruled out in a painful knee after TKA, arthroscopic debridement appeared to be a good option to resolve the pain.

## 1 INTRODUCTON

Total knee arthroplasty (TKA) is one of the most successful surgeries in orthopedic surgery [1-3]. However, from the perspective of patient satisfaction, TKA has been reported to be inferior to total hip arthroplasty (THA) [4]. Patients may complain of knee pain after TKA due to infection or aseptic loosening [2, 5]. Infection and aseptic loosening of the components could be evaluated by plain radiographs, bone scans, clinical findings, serological data, and knee joint aspiration with cytological examination and culture of synovial fluid. For the treatment of infection or aseptic loosening, many cases require revision surgery. On the other hand, if serological data, cytological data of the synovial fluid, and radiological findings are normal, it is not easy way to determine the precise cause of the pain after TKA in many cases. In the literature, 5% to 9% of the patients complained of pain after TKA for no apparent reason [6-9]. Soft tissue impingements of several pathological entities have been reported. Patellar clunk syndrome (PCS) [10-13] and patellar synovial hyperplasia (PSH) [10, 14, 15] are the two main entities of impingement; however, other types of impingement including soft tissue impingement at the intercondylar notch [16], impingement of the posterior cruciate ligament stump [14, 17, 18], and arthrofibrosis [17-20] have also been reported. In these diseases, if the patients are resistant to conservative treatment, arthroscopic surgery is indicated instead of revision surgery in many cases [10, 12, 18]. In the present report, 30 cases of another type of soft tissue impingement different from the above-mentioned soft tissue impingements are described, along with the arthroscopic findings and surgical results.

## 2 MATERIALS

From October 2005 to March 2014, 46 cases of arthroscopic surgeries were performed for patients with pain after TKA for more than 6 months. Of the 46 cases, 16 were excluded due to infection (9 cases), PCS (5 cases), and short follow-up after surgery (2 cases). The remaining 30 cases had moderate to severe medial or lateral joint line tenderness, and they also had pain during walking and/or pain at rest. Of the 30 cases, there were 3 males and 27 females. Their average age at the time of arthroscopic surgery was 72 years (range, 51-86 years). The average times between the index TKA and the onset of the pain or arthroscopic surgery were 18 months (range, 1-144 months) and 29 months (6-125 months), respectively. The average follow-up period after arthroscopic surgery was 36 months (range, 6-93 months). The types of TKA were posterior stabilized (PS) type in 26, cruciate retaining type in 2, cruciate substituting type in 1, and rotating platform type in 1.

### 2.1 Surgical Technique

All surgeries were done by a single surgeon (HS) with the patient under general anesthesia. Three arthroscopic portals were used for complete arthroscopic debridement: anteromedial, anterolateral, and proximal superomedial portal [21]. Viewing through the anterolateral portal, the routine diagnostic arthroscopic examination was performed with a probe inserted through the anteromedial portal. After finishing the diagnostic examination, the proximal superomedial portal was placed 4 cm proximal to the superomedial margin of the patella. Through the portal, the medial gutter, lateral gutter, patellofemoral joint, anterior portion of the medial and lateral tibiofemoral joint, and the complete infrapatellar fat pad were observed. Any scar tissue found at the medial or lateral tibiofemoral joint, patellofemoral joint, intercondylar notch, and posterior aspect of infrapatellar fat pad was removed with an electric shaver and punch. No immobilizer was used after the surgery. One day after the surgery, the patient was allowed walking with full weight-bearing.

## 3 OUTCOME MEASURES

**Fig. (1):**
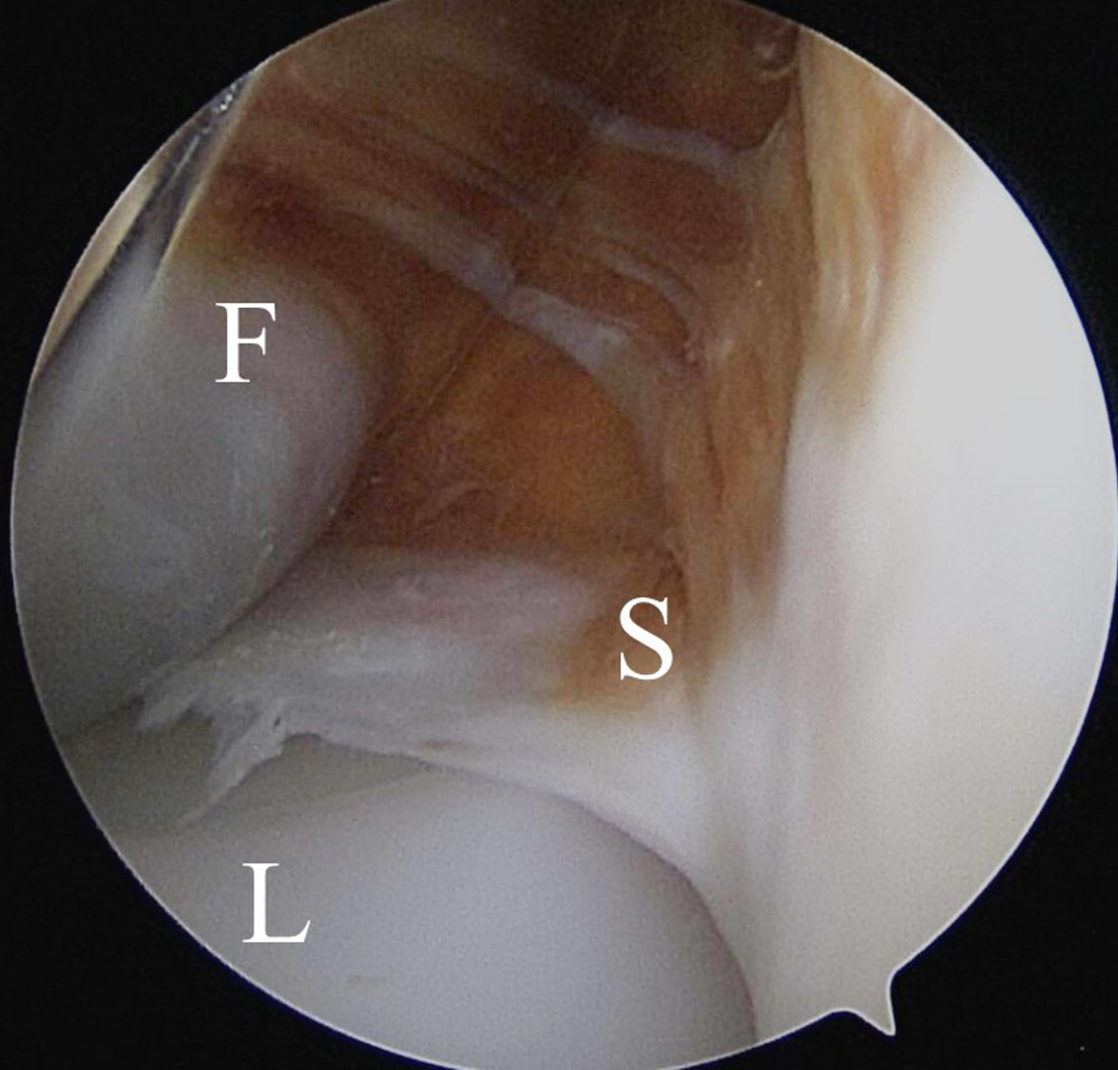
Arthroscopic findings of the medial tibiofemoral joint from the anterolateral portal (right knee). Degree of the scar tissue impingement was classified as small in this case, by the length of the scar tissue over the polyethylene line. The scar tissue was connected with the scar tissue over the fat pad. F: femoral component, L: tibial polyethylene liner, S: fibrous scar tissue.

Based on the arthroscopic findings, synovitis inside the joint, scar tissue impingement, and scar tissue coverage on the infrapatellar fat pad were classified as follows. We classified the degree of synovitis into four types as no, mild, moderate, and severe proliferation. The degrees of scar tissue impingement at the patellofemoral joint, tibiofemoral joint, intercondylar notch were classified into 4 types by the maximal width of the scar tissue beyond the margin of the implant as none (less than 5mm), small (5-7 mm), moderate (8-10 mm), and large (more than10 mm) (Fig. **1**). The infrapatellar fat pads were classified into 3 types by the scar tissue coverage on the fat pad, as no scar tissue, half coverage, and full coverage.

The range of motion and Knee Society Score [22] were evaluated preoperatively and at the time of final follow-up. The degree of pain at final follow-up was classified into 6 types: completely painless, marked improvement, 50% improvement, slight improvement, no change, and aggravated. No cases had complications of infection, symptomatic venous thrombosis, or pulmonary embolism.

## 4 RESULTS

Degrees of the synovitis were no proliferation in 30% and mild in 70%; no case had moderate or severe synovitis. Proportion of the cases graded by soft tissue impingement as moderate or severe were 50% at the patellofemoral joint, 30% at both the medial and lateral tibiofemoral joint, and 77% at the intercondylar notch. Details of soft tissue impingement are shown in Fig. (**2**). In all cases, the infrapatellar fat pad was fully covered with white scar tissue, and the scar tissue at the infrapatellar fat pad had continuity with the neighboring scar tissue found at the tibiofemoral joint.

**Fig. (3):**
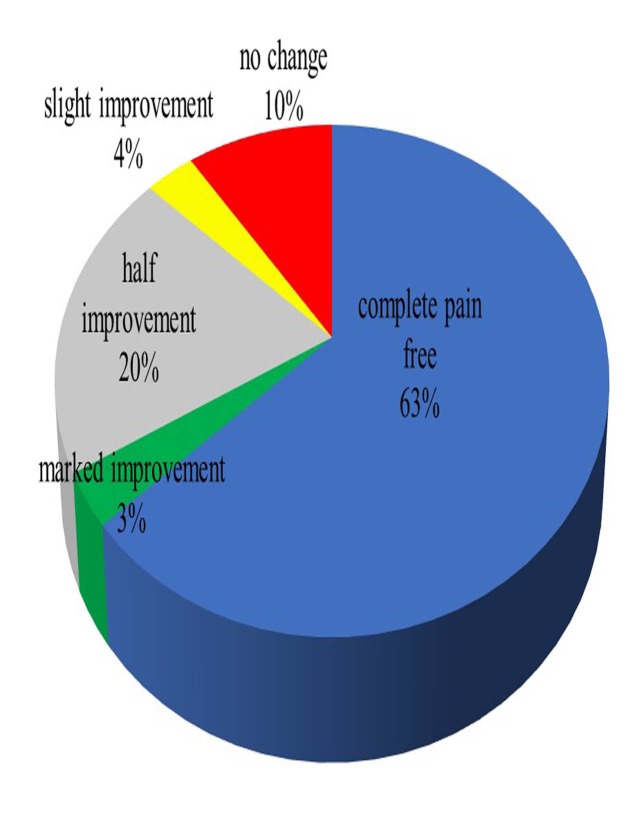
Clinical results at the final follow-up.

The preoperative range of motion was -1.5 to 115.2 degrees, and the postoperative range of motion was -0.6 to 116.8 degrees. The Knee Society Score was 75.1±4.8 points preoperatively, and 86.9±11.3 points postoperatively. Pain status at final follow-up was completely painless in 63%, marked improvement in 3%, 50% improvement in 20%, slight improvement in 3%, no change in 11%, and aggravated in 0% (Fig. **3**).

## 5 DISCUSSION

Following TKA, PCS and PSH are well known painful pathologies that can occur postoperatively. Hosack *et al.* first reported PCS [11]. PCS was traditionally associated with a PS implant. The symptoms occur secondary to the formation of a proliferative, fibrous nodule in the superior pole of the resurfaced patella. The nodule engages the femoral intercondylar box of the PS implant during knee flexion. The clunk represents the dislodging of the nodule from the box during knee extension. The reported incidence of the syndrome has ranged from 0% to 12% [23]. Patients with PCS complain of painful catching or a “clunk” from flexion into knee extension. Arthroscopic treatment of PCS has been widely accepted as successful in terms of symptom resolution and a low recurrence rate [10-13].

Pollock *et al.* reported another type of soft tissue impingement at the patellofemoral joint of posterior stabilized TKA termed “synovial entrapment”, which was named “synovial hyperplasia” in another report [24]. Hypertrophic fibrous tissue proximal to the patella was entrapped during active knee extension from 90 degrees [10]. The patient complained of pain and crepitus, not clunking, when rising from a sitting position or during stair climbing. Pollock *et al.* noted that implant design had an intimate relationship with this phenomenon [24]. These types of impingement were successfully treated by arthroscopic debridement [10, 25].

Bonutti *et al.* reported 19 knees of femoral notch stenosis caused by soft tissue impingement accompanied with late-onset arthrofibrosis after posterior-stabilized TKA [26]. They believed that the hypertrophied soft tissue in the intercondylar notch was irritated by the repetitive motion of the tibial post during knee flexion ante extension. All but one patient had clinical improvement at the final follow-up at a mean of 54 months. The arthroscopic findings of their cases included marked hypertrophic soft tissue in the intercondylar box and a fibrous mass at the infrapatellar fat pad, which prevented full extension. These arthroscopic findings were relatively similar to those of the present cases; however, decreased range of motion was not observed before surgery in the present cases, and the degree of soft tissue hypertrophy was much less in the present cases than in the cases of femoral notch stenosis.

In the present cases, 50% had scar tissue graded as moderate or severe over the patellar component, and 77% of the cases had soft tissue proliferation graded as moderate or severe at the intercondylar notch. However, no case in the present study had tenderness at the patellofemoral joint, a positive patellofemoral grinding test, or clicking or snapping during deep flexion to extension, which are findings of PCS [11] or PSH [10]. On the other hand, all cases had marked tenderness at the medial and/or lateral tibiofemoral joint space. Given these clinical findings, the pathology of the pain in the present cases appeared different from previously reported cases of PSH, PCS, or arthrofibrosis including femoral notch stenosis.

As shown in Fig. (**2**), the size of the soft tissue that impinged between the femoral component and the tibial component was relatively small in 70% of the cases. Nevertheless, most patients complained of marked tenderness at the tibiofemoral joint line. Steadman *et al.* [27] reported symptomatic scarring of the anterior interval of the knee that was defined as the space between the infrapatellar fat pad and patellar tendon anteriorly, and the anterior border of the tibia and the transverse meniscal ligament posteriorly. Scarring of the interval causes decreased excursion of the patellar tendon in relation to the anterior tibia may lead to the anterior knee pain. And, they reported successful results of arthroscopic release of the interval for 25 symptomatic cases by reducing the pain. We speculated that the similar scarring could be the cause of the impingement found in our cases. All soft tissue that impinged at the tibiofemoral joint space had less mobility due to continuity with the scar tissue covering the infrapatellar fat pad. The decreased mobility of the soft tissue must be the reason for impingement, even though the impinged tissue was small.

To cut out the connection between the infrapatellar fat pad and the scar tissue at the tibiofemoral joint, it was decided to remove all soft tissue at the tibiofemoral joint space and over the infrapatellar fat pad. It was very difficult to perform these procedures from the anterior portals, because these structures were very close to the anterior portals. The proximal superomedial portal [21] was convenient enough to observe all structures, and it was possible to use the anteromedial or anterolateral portal as the working portal to insert the electric shaver or other devices to remove the scar tissue.

This study had several limitations. First, it was a retrospective study, and all cases were symptomatic. For accurate evaluation of the arthroscopic findings in this study, they should be compared with those of asymptomatic cases after TKA. However, it would be difficult to perform arthroscopy for healthy asymptomatic knees from the ethical perspective. Second, it was not possible to evaluate the importance of removal of scar tissue that had covered the infrapatellar fat pad, because the procedure was performed in all cases. Third, the possibility of recurrence of scar tissue formation could not be ruled out, because second-look arthroscopy was not performed in any case.

Of the 30 cases, 21 underwent the index TKA at our institute. These cases account for 4.3% of the total 487 TKAs over that period in our institute. Posterior-stabilized TKA (Scorpio NRG, Stryker Orthopaedics, Mahwah, NJ, USA) was used in all cases in the period. However, it is not possible to determine whether the soft tissue impingement observed in the present cases was predominantly a complication of PS TKA. Nevertheless, when we perform TKA, especially PS TKA, we should be aware that this type of soft-tissue impingement can occur not infrequently.

## CONCLUSION

Soft tissue impingement at the tibiofemoral joint that was different from previously reported pathologies, such as PSH, PCS, or arthrofibrosis including femoral notch stenosis, was described. The characteristic sign of the impingement was marked tenderness at the tibiofemoral joint space. When conservative treatment was unsuccessful, arthroscopic debridement had satisfactory results in many cases.

## Figures and Tables

**Fig. (2):**
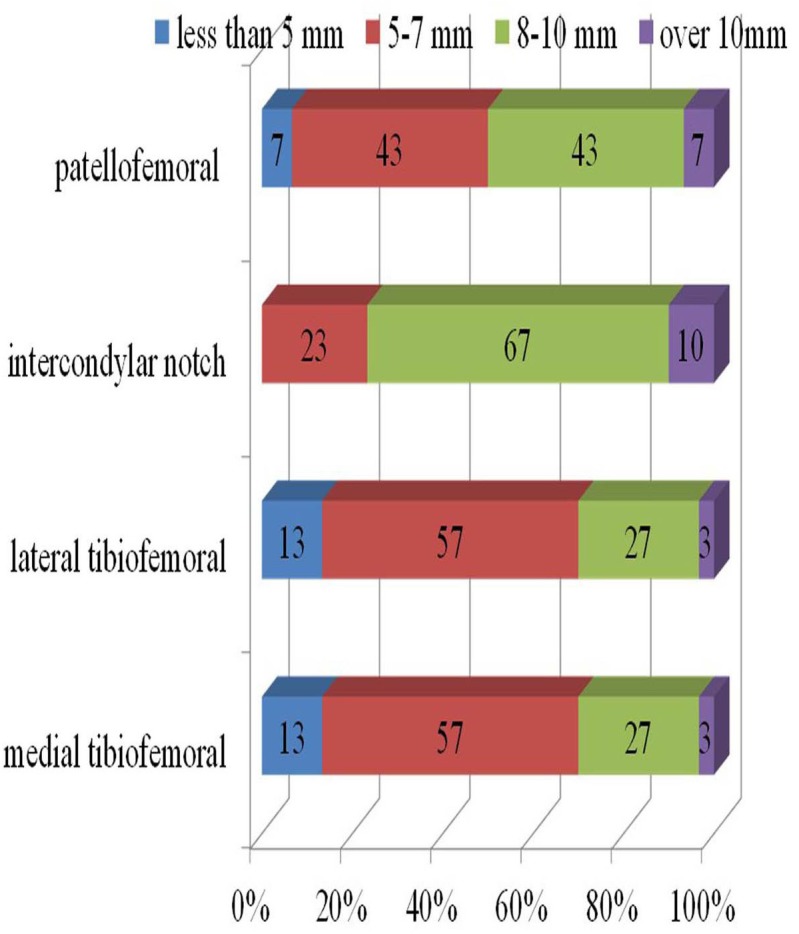
Size of fibrous scar tissue impinged at each compartment.

## LIST OF ABBREVIATIONS

PCS: = patellar clunk syndrome
PSH: = patellar synovial hyperplasia
THA: = total hip arthroplasty
TKA: = total knee arthroplasty
